# Gatekeepers of Reward: a Pilot Study on the Ethics of Editing and Competing Evaluations of Value

**DOI:** 10.1007/s10805-018-9305-6

**Published:** 2018-04-16

**Authors:** David M. Shaw, Bart Penders

**Affiliations:** 1 Department of Health, Ethics & Society; Care and Public Health Research Institute (Caphri), Maastricht University, PO Box 616, Maastricht, Limburg, 6200MD, the Netherlands; 2 Institute for Biomedical Ethics, University of Basel, Basel, Switzerland

**Keywords:** Editorship, Valuation, Value systems, Reward

## Abstract

The reward infrastructure in science centres on publication, in which journal editors play a key role. Reward distribution hinges on value assessments performed by editors, who draw from plural value systems to judge manuscripts. This conceptual paper examines the numerous biases and other factors that affect editorial decisions. Hybrid and often conflicting value systems contribute to an infrastructure in which editors manage reward through editorial review, commissioned commentaries and reviews and weighing of peer review judgments. Taken together, these systems and processes push the editor into a role resembling censorship. Editors and authors both experience this phenomenon as an unintended side-effect of the reward infrastructure in science. To work towards a more constructive editor-author relationship, we propose a conversation, an exchange between editor and author in which value is collectively assessed (or constructed) as obligatory passage points in the publishing process are traversed. This paper contributes to the discourse on editorial practices by problematising editorial paradigms in a new way and suggesting solutions to entrenched problems.

## Introduction

The production and distribution of scientific credit, responsibility, and accountability has garnered growing attention amongst those pursuing critical enquiries into the epistemic and social organisation of science and the consequences for the knowledge produced as a result (Hackett et al. 2017). The studies conducted vary in their focal points, ranging from authorship cultures (Biagioli 2003; Marušić et al. 2011; Youtie and Bozeman 2014), citation cultures (Shrum 2010; Wouters 1999), the use, abuse and performativity of metrics (Hicks et al. 2015; Rushforth and de Rijcke 2015), the organisation, quality and consequences of peer review (Bornmann and Daniel 2010; Schwartzman 1997) to the online construction of the self by academics (Fochler and de Rijcke 2017; Hammarfelt et al. 2016). Combined, they provide an ever-growing understanding of research practices and the attribution and evaluation of value in those practices. However, despite these riches, the study of scientific credit, responsibility, and accountability continues to be limited to the study of academics as authors and peer reviewers (and research managers to a lesser extent). The role, responsibility and burden of the editor has been left largely unstudied.

With scientific credit, responsibility, and accountability being located increasingly in the publishing process, this makes the journal editor a gatekeeper in the assignment, attribution or construction of scientific merit, credit and value - acting as a gatekeeper in science’s culture of reward. Rewards, in the form of credit, recognition and prestige, form the dominant scientific currency (Collins 1975; Latour and Woolgar 1979). In this paper, we provide a pilot analysis of such gatekeeping practices, in which we diagnose the competing value systems that converge on editors and the ethical issues that emerge from this competition. For the purposes of this paper, we will reflect upon editorial decision-making, editorial review and editorial commissioning, while recognising that active editorship amounts to more than only these aspects. Editorial decision-making includes rejection of papers *before* peer review, often based on criteria unknown to authors; it includes selection of reviewers who may be unfamiliar with or unsympathetic to the content of a given paper; it also includes decisions *following* peer review that may seem to contradict reviewers’ verdicts, and sometimes rejection is for a reason that could have been given before seeking external review. In this empirically motivated reflection, we draw from our own real-life encounters with editors and aim to reconstruct some of the elements of the politics of editorship from interactions that have occurred over the years.

We will first explore existing theory, however limited, on valuation and editorship. Second, we will discuss the notion of ‘internal’ editorial review, problematising the process based upon the conflicting value systems within which it occurs. Third, we dive into the commissioning of articles before concluding with a number of reflections on problematic encounters between editors and valuation cultures.

## Conflicting Value Systems and Editorship

Research on editorship is scarce. Hirschauer (2010) has conducted an ethnography of editorial decisions and has described them as *judgements of judgements*, editorial judgements of peer review judgements, in which criticism is organised and made visible. The selection flowing from such judgments has been described as ‘voting’ (Hirschauer 2010) or represent editorial decision making as ‘a sieve’ (Maurer 2013), ‘employing a host of editorial values’ (Corlett 2009) – each a form of gate keeping and of evaluating and assigning value. However, such judgements are not immune to interests or misconduct, as a series of studies have demonstrated (da Silva and Al-Khatib 2016; Gottlieb and Bressler 2017; Teixeira and Da Costa 2010).

More prominent are studies on the role, political context and history of the scientific journal, in which editors occasionally feature prominently. Franzen (2009), for instance, argues that journals serve two cultural goals. The first and most obvious is to cater to the dissemination needs of researchers. The second goal is to act as a windows through which a professionalised public (including, but not limited to science writers and science journalists) looks at science. While the first goal is easily recognisable across the board, the second one is more overt for the ‘big journals’ including *Science, Nature, Cell, PNAS*, and a few others, while it seems obscure for many others. However, even the automated distribution of tables of content to peers already provides a modest indication of what Franzen and colleagues call the *medialisation of science* (Franzen 2009; Franzen and Rödder 2013; Franzen et al. 2012; Weingart 2012). Mass-medialisation of science comes with a risk. Franzen argues that “As multidisciplinary science media, built upon a latent sensitivity towards mass medial selection criteria, Science and Nature are in danger of subordinating scientific relevance to mass medial relevance” (Franzen 2009 p. 229). In other words, in the context of mass-medialisation of science, a second value system for deciding on the quality, merit and value of contributions has come to the fore – competing with the scientific value system we assume to exist. Especially in the context of the ‘big journals’, the way in which they establish themselves in the face of scientific and media scrutiny is thus subject to two potentially and probably very conflicting sets of criteria. Choices regarding contributions are not made solely on the relevance of their content, but also on whether they fit media expectations (e.g. Weingart 1998). The same goes for the selection of editorials and other front matter that is weighed against expectations of external perceptions of e.g. media, which are important allies to then ‘big journals’ to maintain their scientific and media status.

However, journals are not mere infrastructures managing reward and knowledge flows, coproducing them through selection. Scientific journals, as well as their editors, cannot be separated from their status as outlets of large publishing houses or their image and earning potential to responsible associations. The fact that publishing is a business means that editors are subject not only to academic and research agendas, but also to commercial imperatives. Already in 2013, only a handful of companies were responsible for the production of roughly half of all research papers (Larivière et al. 2015). Given the sheer number of articles flowing through editor’s offices, complex infrastructures have been set up to ‘assist’ the editor in managing the flows. In the context of journals as commercial entities, Taubert (2012) has argued that these so called ‘online editorial management systems’ contribute to an organisation of editorial work, including infrastructures for control, typical for highly formalised organisations. This, he argues, is indicative of the expansion of the publisher’s power into the offices and minds of editors, and to a lesser degree even authors and reviewers. In the most radical sense, Taubert argues that the boundaries of the publisher have expanded into the academic domain causing “a clash of scientific and economic rationalities” (Taubert 2012 p. 297). Following Franzen and Taubert, this allows us to view editors, their work and the context of their work, as assigning value to manuscripts, and basing decisions about them, on at least three distinct, competing value systems: scientific, medial, and economic.

## Methodological Note

This is not a typical empirical paper. We include descriptions of interactions with editors as examples of the types of issues that may arise and present an ethical discussion of the effects of competing and conflicting value systems as they converge on editors. As such, our paper provides an ethical analysis informed by real-world stories. We have chosen to exclusively use our own interactions with editors of journals in medicine and the life sciences. Communication between editors and authors does not take place in the public domain and while we could have attempted to collect these interactions, we would have no way to validate whether we were handed a carefully selected subset (either by editors or by authors). We realise that the topics we deal with in our own line of research, including but not limited to research integrity, publication ethics, the sociology of scientific knowledge and science and technology studies, predisposes us to critical interactions with editors. If anything, this may serve to make valuation dynamics more overt. However, we are very aware that we are presenting a pilot analysis, rather than a fully developed ethics or sociology of editorship.

We cannot, however, provide *ad verbatim* testimonies by the editors we interacted with. Communications between editors and authors are private, as indicated by journals’ terms and conditions and are in some cases explicitly included in every email message: “This e-mail is confidential and subject to copyright. Any unauthorised use or disclosure of its contents is prohibited”. Therefore, rather than contacting editors and risking their refusal to use direct quotations, we paraphrased all the interactions we include below and removed all indicators that may reveal the editor’s, journal’s or publisher’s name. Our paraphrasing preserves the original meaning of editors’ statements although the wording has been changed. This is not the case for public statements or quotes drawn from the public domain. In the context of our pilot analysis this is not an immediate problem, since we seek to initiate a structural understanding of how editors assign value and decide accordingly. Our interactions with editors are not generalisable in an empirical sense, but the lessons we draw from them may inform editor-author interaction around the value or worth of scholarly work in the future.

## Initial Internal Review

Editorial review and the ways in which it shapes publishing are most visible in situations where peer review is either absent or less important. Consider, for instance, large multidisciplinary journals which mainly publish original scientific research, yet for various (political) reasons also publish significant amounts of front matter, or ‘commentary-type’ articles. While it is true their focus is on original scientific research, which is peer-reviewed, these journals tend to review and reject many submissions internally without sending them out for review. Such decisions can be the result of considerations of quality, but they may also occur because the editors disagree with the content despite recognising its relevance and potential impact. A case in point was when one of the current authors had two rejections from a major science journal because editors disagreed with the conclusions of the content. In the first of these, a commentary discussing alternative authorship cultures, the reason for rejection was, paraphrased, as follows:

Your arguments in favor of open review and anonymous authorship are both interesting and thought-provoking. I agree that there lies value in a system in which people sign their names alongside their opinions. However, there is also value in a system in which people are free to speak openly without fear for personal consequences. In sum, I am not convinced that the bias and power redistribution your suggestion produces are smaller than the ones currently in place. Entirely anonymous authorship, while possibly attractive, is very impractical. I regret that we cannot consider publication of your paper.

Here, the editor is essentially admitting that the paper is not being sent for review because the editor does not find the arguments convincing, despite seeing merit in them. The fact that her position might be due in large part to value systems promoting the current system within which she operates is not mentioned. Shutting down debate in this way, editors do a disservice to the scientific community.^1^ In response to another paper, the same editor replied (paraphrased):

We accept that there are valid arguments towards avoiding effort by peer reviewers, and we do work towards this by sharing reviews between journals at X, as you already indicate. However, we are unconvinced that an online database containing all reviews would yield more benefit than harm, given the current publishing model.

Here, the reason given is again that the editors do not find the arguments convincing – a judgment ordinarily left to peer reviewers. More specifically, the editor inadvertently concedes that the arguments in the paper point towards changing the model of publishing – again challenging the value systems at play.^2^

In both these cases, the editors’ unilateral rejection is particularly ironic given that both manuscripts suggested changes in standard editorial practices. Would they have done this with original research, even if it challenged traditional scientific paradigms? Presumably not, given that the big journals prize novelty – sometimes to a fault, itself testimony to a medial and economic value system being at play. This attitude apparently does not extend to anyone daring to suggest that current editorial paradigms should change. And those current paradigms can be quite exacting. An unnamed intensive care journal rejected one of our papers with the following paraphrased response:

To ensure efficient review and publication, we have instituted a policy stating that only those submissions that have the approval of all editors are forwarded to the next stage. This policy aims to provide authors with a quick response and avoid delays in case you want to submit your manuscript to a different journal.

This effectively means that any paper must meet with the unanimous approval of all editors before even being sent out for peer review. This particular manuscript was suggesting a controversial clinical technique for organ preservation – one unlikely to meet with approval from all editors because of its challenging ethical nature. Allowing each editor a veto over controversial content is likely to stifle innovative ideas. Applying such a policy to the more standard original research articles this journal publishes, would stifle innovative thought, and, by default, disqualify potentially paradigm-shifting work. Next to the policy itself, ethical questions arise on how it came into being, what inspired it, and how such a decision ought to be communicated to potential authors.

Editorial review thus actively and overtly politically preselects the contributions that move on to the second round. Such selection does not only take place via the rejection of manuscripts. Even when manuscripts are willingly and enthusiastically received by an editor, the editor may choose to continue to influence their content. Consider, for instance, an original research manuscript submitted by one of us, which solicited a response requiring a specific rewriting before it was to advance to the next stage: “I do like your topic and general approach. I hope that you will consider expanding this paper –some suggestions are below […]”. The suggestions that followed took up over 2000 words excluding a long list of specific literature to be included. Of course, journal editors are, in most cases, experts in their own right. Their expertise may be equally relevant to the manuscript discussed, as that of peer reviewers. Key differences lie in the coverage of their input (potentially all papers in their journal) and the power that accompanies it, allowing a body of literature to be ‘tweaked’ even before its quality or value can be evaluated through peer review.

The editor of the *New England Journal of Medicine* has suggested in an interview that “peer review is only a first step to vetting papers that may be interesting and relevant for readers. After a paper passes peer review, it is then given to a team of staff editors who each have a lot of time and space to go through the submission with a fine-toothed comb. So highly qualified editors, not necessarily peer review, act as the journal’s gatekeepers​” (Belluz and Hoffman 2015). This is unquestionably a valuable process, provided the editors are scrupulously fair and only apply their skills post-review. But if these editors reject many papers despite positive reviews, perhaps such a system may waste the time of both peer reviewers and authors (see below). In many cases, however, editorial review precedes peer review.

In this way, editorial review prior to peer review is shaping the flow of ideas and concepts into epistemic cultures one decision at a time. Control of this flow, however modest, is hardly ever identified as such, and it ranges from rather obvious cases to very subtle ones where editors might be totally unaware of what they are doing. Control of this flow may even in some cases amount to a modest form of censorship, however small, when ideas that challenge publication practices (as described above) are concerned. We are not the first to suggest that editorial control can amount to censorship. For instance, “Biagioli ties the establishment of editorial peer review to the royal license that was required for the legal sale of printed texts” (Fitzpatrick 2011 p. 21). It allowed the academies to have their members ‘peruse’ books before print, which Biagioli argues was about censorship much more than quality: “As in traditional book licensing, the review was about making sure that a text did not make unacceptable claims, rather than to certify that it made good claims” (Biagioli 2002). In terms of impact or scale, current semi-censorship is much more plural, but hardly absent.

One effect of awareness of this process among authors is that they may attempt to self-censor.^3^ Based upon previous interactions with journals and editors, and experiences shared by our peers, we ourselves carefully select which journal to send our ideas to, and how to package them. This is effectively internalising the competing valuation practices into self-censorship, reinforcing both research paradigms dominant in journals, and publishing paradigms dominant at journals (and publishers). There are many thousands of journals and most indicate with some clarity the scope of work they seek. Of course, a receptive destination can normally be found (for example) for a theoretical analysis in a bioethics journal if a medical journal will not accept it. However, as we argue, the decision not to accept need not (solely) be the result of limits to the episteme occupied by the journal. As a consequence we must ask if it is questionable whether authors should be obliged to target entirely different readerships because of the entrenched editorial practices, struggling with competing evaluations of the worth of scholarly contributions, at one journal.

## Commissioned Articles

Through editorial review and the rejection of manuscripts, or influence on their content, editors shape the epistemic content of their publication outlets, as well as explicitly withholding or granting scientific and scholarly reward accordingly. In order to affect such influence, editors are not limited to passively awaiting new submissions. While original research articles can be submitted by anyone, many of the commentary, perspective or opinion pieces – as well as a large number of (meta-, systematic- or other) reviews at many journals are, in part or in full, commission-only. Editors have the liberty to actively seek out ideas and to actively construct reward and value amongst those ideas.

We have in mind not so much the commentaries that often accompany research articles (and which thus must be commissioned) but standalone Comment pieces labelled ‘Opinion’, ‘Commentary’, ‘Viewpoint’ or otherwise and that can be found in many journals. For example, in Nature’s instructions for authors, it is stated that “Unsolicited contributions are not accepted. This is a commission-only section intended to be accessible and appealing to the whole global Nature readership, of all disciplines.”^4^ Here, it is explicitly stated that such papers cannot be submitted unless commissioned; this is rather ironic given that “Comment pieces are generally agenda-setting, authoritative, informed and often provocative expert pieces calling for action on topical issues pertaining to scientific research and its political, ethical and social ramifications.” This means that Nature’s specific category for agenda-setting and considering ethical and social ramifications of research is off-limits to open submissions – and it also means that only editors can select who gets to set the agenda.

These options further expand the epistemic and political reach of editors – as well as imposing aforementioned value systems upon contributors of these texts, while obscuring them from the readers of the same texts. We can discern three routes by which the epistemic and political reach of editors exerts itself. First, through commission-only contributions they are narrowing the ability of researchers themselves to set the agenda of their field. They will have fewer pages per issue available for original contributions in which they may articulate their own ideas (provided free of editorial review) and have to compete with other genres of contributions. Furthermore, if commentary-type articles are the only or preferred way to get an original idea that is not a typical piece of research into a major journal, then making such pieces commission-only in effect shuts out all other authors, obscuring the status or spread of that idea in the community. Second, commissioned pieces are articulations of the editors’ agenda, meaning that a commissioned piece is very unlikely to take a critical stance towards paradigms dominant in that journal. Third and finally, even if a commissioned writer chooses to challenge orthodoxy, forward novel and critical ideas or provide potentially paradigm-altering work, he or she might find it difficult to persist if the editor who commissioned the piece prefers a different view.

Commission-only articles act in tandem with the aforementioned semi-censorship of ideas by editors; on the one hand, editors censor ideas that challenge current scientific or publishing paradigms, while on the other, they commission pieces that promote and extol the virtues of the current dominant paradigms (while also depriving authors of the ability to contribute opinion-type pieces). One example occurred when one of us had an article accepted by a major medical journal, and the editors commissioned an editorial from a politician who appropriated the key concept from the article and used a misunderstanding of it to campaign for a change in organ donation systems. The editors did this without informing the author and creator of the concept in question, who could have pointed out to them prior to publication the problems with the editorial. To their credit, the editors apologised when a complaint was made, but the damage was already done.

## Post-Peer Review Decisions

In addition to epistemic control through preselection and commissioning and influence through pre-review, editors have further tools available to construct specific contents of their journals and distributing the value and rewards associated with them. When a paper is submitted to a journal and subsequently sent out for peer review, editors will be on the receiving end of those reviews. In our considerable experience as authors of over 100 papers, rather than merely forwarding them, editors almost always attach judgements to the set of reviews. This provides assistance to authors, who have to navigate difficult value judgments by reviewers. For instance, if reviews contradict one another in terms of content, an editorial comment may indicate which review the editor considers dominant. If reviews fail to raise a specific issue, the editor may choose to include it nonetheless. If reviews contain different valuations, editors help prioritise them. We observed that in some cases editors themselves act as an informal third or fourth reviewer, making points that neither reviewer mentioned, but which nonetheless must be addressed in order for a paper to be accepted.

Editors may disregard or expand reviewers’ recommendations, and while editors are obviously not obligated to follow reviewers’ suggestions, some editorial decisions very clearly contradict the recommendation without sufficient– or indeed any –rationale. Despite endless and valid critique, peer review is justified by arguments that it maintains quality (e.g. Armstrong 2007). If the value system targeting assessment of manuscripts in the context of scientific or scholarly quality takes precedence over other value systems, this would translate into the position that reviewers should only be ignored under special circumstances. A case in point is a recent interaction one of us had with an editor from a major medical journal. The submitted manuscript had been reviewed and two reviews were forwarded, as well as the editor’s decision. The first review began with a sentence paraphrased as “A highly relevant and clearly important paper which all medical professionals should read” and concluded with “The manuscript allows as much depth as possible. All arguments are sound, logical and well-articulated.” Reviewer 2 was also enthusiastic, paraphrased as: “the argument and analysis are well-developed and significant”. However, the editor rejected the paper and suggested cutting it to 700 words and submitting it as a blog instead. Again, this would presumably not have happened were the submission an original research paper reporting a clinical trial.

In another similar case, a review paper on the ‘family overrule’ of organ donation was submitted to a leading transplant journal. Reviewer 1 concluded that the principles articulated in the article were important, it read well and contributed to the current literature on this topic; and reviewer 2 opined, paraphrased, that

This is a balanced text, providing reasons both for and against family overrule. It is comprehensive as well as gives pertinent ethical recommendations and reasoning when dealing with familial objections to organ donation. Overall, a well written manuscript.

However, the editors rejected the paper, giving the following reasons: “This non-quantitative manuscript is interesting, yet does not provide clear recommendations rooted in scientific data. Accordingly, the manuscript is better suited for an ethics journal.”

When this decision was appealed on the grounds of the very positive reviews, the editors rejected the appeal on the grounds that in addition to the comments to the authors, the reviewers also scored the manuscript on novelty, significance and impact. Editors argued that the manuscript was assigned moderate scores by both reviewers, which informed the decision not to accept the paper. Given the favourable reviews and the fact that these scores were not provided to the authors, this explanation is far from convincing. A more plausible explanation is that the editor considers non-quantitative research to be of less value. Moving beyond examples drawn from our own interactions with editors, in extreme cases, post-review editorial decisions can work in the opposite direction. Rather than rejecting critical work, competing value systems can convince editors to accept papers that are highly media-savvy, filled with controversy and correspondingly score well on various metrics, despite being rejected by peer reviewers based on research quality assessments. Only one such instance is concretely known to us, namely the paper ‘The case for colonialism’ by Bruce Gilley, in which competing evaluations of worth have become very visible beyond editorial offices and have, accordingly, created extensive debate that contributed to the resignation of half of the journal’s editorial board and ultimately, the retraction of the paper.^5^

## Discussion

In this paper, we attempt to initiate an ethical mapping of the role of editors in science’s culture of reward. The narratives we chose to paraphrase illustrate an under-recognised political role for the editor, that of the censor. It is through semi-censorship that editors grant or withhold reward; it is through semi-censorship that the epistemic content of journals is constructed and through semi-censorship that the status and credit of scientists is decided upon.

When we talk of censorship, we do not refer to explicit, premeditated, politically planned and governed control of information flows. Of course, many journals are specifically tailored to specific schools of thought in a discipline and have, as such, an identity drawn from the epistemic paradigm they subscribe to. Through their decisions and action, editors guard this identity. This is a process that is visible from the outside, and known amongst the group of authors the journal targets. It usually can be understood via consulting journal websites and guidelines. As far as selection is in the service of this identity, it falls short of being labeled censorship. However, to avoid dogma and the dismissal of the potential value of critique, these paradigms can never be considered as being above discussion.^6^

In practice, the censorship as conducted by editors will be implicit, unknown and very probably invisible to most – including authors and the editors themselves. Some editors are among the victims of this particular social, political and economic organisation of science. Editors handle large numbers of manuscripts, whether original research or other types, and value judgements are required for each and every one of them. These decisions cannot be separated from the value systems in which the editors are embedded themselves. Editors judge based upon scholarly value, medial value, economic value and probably many other values, and hybrids of all of them. Again, these value systems and especially intermediaries or hybrids are implicit and unknown. Except for a few epistemic preferences, they are not on the journal’s website, they are not in the editor’s manual, yet they are embedded in editorship practices, shaping the distribution of reward in science.

Authors are also victims. In the most visible sense, (primarily) the major, monolithic journals discriminate against theoretical and conceptual articles by making them commission-only, but more importantly by rejecting critical ideas before peer review merely because editors disagree with the content. If they did this with original articles, imagine the controversy: editors reject results of study because they disagree! However, more subtle censorship infrastructures exist through careful nudging of the content of papers through translation of peer review results, additional editorial review steps (pre- or post-peer review), or authors anticipating all of the above and engaging in self-censorship to navigate the conflicting value systems of the contemporary publication landscape.

Of course, the degree to which such semi-censorship is performed will vary greatly across disciplines, journals and regions. The stronger the position one value system is in place vis-à-vis the others, the more it will determine the content of value judgements made by editors. Following Franzen (2009), we hypothesise the ‘top-tier’ journals to be more at risk than any others because of their complex relationship with research success, research excellence, research dissemination and prestige in general (Reich 2013). If these journals (and others) want to remain interdisciplinary and act as a forum for novelty, quality and paradigm-altering contributions, any form, however light, of censorship is a handicap at best. In order to allow value to be granted to a wide array of research types, editors require room for maneuvering which conflicting value systems may withhold. The incentives associated with specific publication outlets and publication types – for acquisition, career development, and building prestige, exemplified in the dominant striving for excellence, further reinforce current censorship practices (Halffman and Radder 2015).

This pilot analysis of editors as gatekeepers of reward has several limitations. First, apart from the literature we cite, it is built entirely on our own personal experiences of interacting as authors with editors (and in the case of DS, interacting with authors as an editor). However, this experience is not inconsiderable, and we have attempted to avoid having any personal biases skew our analysis. This is a pilot analysis using our own data, and we suggest that larger analyses using a wider pool of data would be beneficial and, accordingly invite others to join the conversation. Second, we have paraphrased communications from editors rather than quoting them verbatim in order to respect both their confidentiality and the rules of the journals concerned. Despite our attempts to paraphrase carefully, this limits the reader’s ability to assess the meanings of those communications themselves.

### Recommendations

Editorial valuation practices consist of work, the type of work that goes into assessing contributions, articulating value associated with them internally and translating it to a value judgement that also has merit externally. We propose that editorship, in close allegiance with authorship, should pursue value through dialogue. In practice, this should take the shape of a conversation, a practice in which value is constructed in a manuscript through a series of open interactions in which editorial decisions are less unidirectional decisions and more collective obligatory passage points on the route to publication (and reward) (Vora and Boellstorff 2012). A label we all know, for instance “major revisions required”, would become the collective decision of authors, reviewers and editors, only to be followed by the next obligatory passage point “minor revisions required”, etc. Helgesson and Muniesa (2014) have – ironically, in an editorial – pleaded for such a route.^7^ Rather than call for the consolidation of value systems or abolishing all but one, which would require a large-scale and radical structural rearrangement in the organisation of science, we argue that they should feature prominently and transparently in editor-author conversations and become embedded in both manuscript and judgment of that manuscript.^8^ While there have already been several promising steps towards open peer review, open editorial review is equally important yet much less developed, possibly because editors are more powerful than peer reviewers.

We realise that this call is ambitious, as it requires a re-articulation of the roles of both editors and authors, however small. After an initial adjustment period, such conversation need not entail significantly more work that existing editor-author communication. Existing research on publishing cultures, publishing practices and peer review provide the initial ingredients for such re-articulations. Also promising is the announcement that the American Sociological Association (ASA) is opening up its archives of editor-author interactions.^9^ This would be the place where best practices of the editorial conversation can be identified more concretely. One of us is a senior editor at an ethics journal and has first-hand experience of making such aforementioned judgements, subject to the same value systems, in pursuit of said conversations. Because of its nature as a humanities and social sciences journal, the editorial team members may have a more explicit understanding of the role of challenging paradigms – or displaying their multitude – in the advancement of research. As a result, the journal seldom rejects before review. Nonetheless, even as a member of the editorial team, it has been difficult to persuade other editors to move to a system of blinded review, because of a (mis)conception of at least one other editor that there is nothing problematic about revealing author identities to reviewers. This suggests that editorial traditions themselves are perhaps those most resistant to change, even when supported by some editors. Hopefully the slow transition from being gatekeepers of reward to being more transparent keymasters of collaboration in publishing can now begin.

More research is needed on the various cultures and paradigms that can bias editorial decisions and lead to conscious or accidental censorship. Specifically, we envision three routes towards expanding our empirical and conceptual understanding of editorship and evaluations of value. First, an empirical analysis of the recently opened ASA archives (as mentioned above) would offer clear empirical examples of author-editor conversations. The ASA archives offer the type of data required for a deeper and more constructive understanding of value creation and value evaluation by authors and editors. Second, a larger survey of authors’ experiences of editors acting as gatekeepers and censors, ideally conducted by independent researchers whose experiences are not included in the analysis, would be beneficial. Any such study would avoid the potential for bias in our pilot analysis while also providing more generalisable data. Third, an ethnography of editors focused on the many value judgements that take place in their practices would allow access to the tacit expertise editors build and transfer, how their relationships to publishers influences these, and the articulations they offer themselves when it comes to their gatekeeping endeavours.

In addition, editors should be assisted to assess and avoid (where possible) the potential for dominant paradigms to bias their decisions. Since editors perform their duties largely without any formal training (Galipeau et al. 2016), this would require at least some education about publication paradigms, dogmas and power distributions as part of existing work directed towards publication ethics. At the very least, editors should be made aware that they are operating within a number of paradigms, some with respect to the epistemic content of their discipline and journal, some with respect to publishing practices. Ideally, publishers, journals and editors disclose these for all to see and to take into account upon submitting work. As a consequence, editors may be subconsciously censoring any work that does not agree with the current paradigms to which they subscribe. Of course, this is not a responsibility limited to editors. In the pursuit of editor-author conversations as part of the publishing process, editors, publishers, authors and reviewers should all be aware of the various factors and pressures that affect editors.

## Footnotes

1 While such interactions do provide frustration, we would like to stress that our contribution here is to the debate on reward and credit. We acknowledge that there will be certain cases in which rejection before review is appropriate, but would argue that editorial disagreement with arguments is normally insufficient grounds for rejection.

2 In analysis and theory papers the arguments themselves constitute the research. While editors of social science, philosophy and ethics journals recognise this, science journal editors do not appear to do so. If they did, it is possible that fewer such papers would be rejected without review.

3 We are subject to this pressure as well. An example: we seriously considered using the euphemism ‘epistemic flow control’ in order not to upset editors evaluating this paper, but after realising the irony, opted for (semi)censorship.

4 Source: “Nature, Author Instructions <Other types of Submission>” at https://www.nature.com/nature/for-authors/other-subs

5 See Roelofs and Gallien’s (2017) contribution to the LSE Impact Blog “Clickbait and impact: how academia has been hacked” at http://blogs.lse.ac.uk/impactofsocialsciences/2017/09/19/clickbait-and-impact-how-academia-has-been-hacked/ for a more detailed discussion.

6 This is of course also the reason why in the current publication landscape paradigm-busting publications have difficulties finding outlets.

7 However, their journal, Valuation Studies, is exceptional in its reflective ability to work in and with value systems, to make them explicit and offer heuristics to understand decision-making in the context of valuation.

8 Making explicit all valuation paths that occupy editorial decisions on journal websites would be equally difficult since they are often tacit. As with all tacit knowledge, it becomes visible more easily when challenged, even by something as simple as a brief reciprocal conversation.

9 See http://www.asanet.org/news-events/asa-news/give-permission-use-your-work-digital-archives
